# 108. Evaluation of the Impact of Dalbavancin Usage on Clinical Outcomes, Cost-Savings, and Adherence at a Large Safety Net Hospital

**DOI:** 10.1093/ofid/ofab466.310

**Published:** 2021-12-04

**Authors:** Wenjing Wei, Norman Mang, Jessica Ortwine, Jessica A Meisner, Richard Lueking

**Affiliations:** 1 Parkland Health & Hospital System, Dallas, TX; 2 University of Pennsylvania, Philadelphia, PA; 3 UTSW, Dallas, Texas

## Abstract

**Background:**

Dalbavancin is a long-acting second-generation lipoglycopeptide antibiotic with potent activity against Gram-positive organisms. Dalbavancin is currently FDA approved for acute bacterial skin and soft tissue infections (ABSSTIs). Growing evidence suggests that patients can be successfully treated with dalbavancin for indications outside of skin and soft tissue infections which include bacteremia and osteomyelitis (OM) with significant cost savings and reduced length of stay. We developed a protocol for the use of dalbavancin in patients who required intravenous antibiotics for serious bacterial infections but did not qualify for outpatient parenteral antibiotic therapy (OPAT). During the COVID-19 pandemic, we expanded the protocol to reduce the amount of clinical contact required for all patients.

**Methods:**

In this retrospective observational study, we reviewed all patients that received at least one dose of dalbavancin in either inpatient or outpatient setting at Parkland Hospital from July 2019 through February 2021. Patient demographics, type of infection, and rationale for dalbavancin were collected at baseline. Clinical response was measured by avoidance of Emergency Department (ED) visits or hospital readmission at 30, 60, and 90 days. In addition, a separate analysis was conducted to estimate hospital, rehabilitation, or nursing home days saved based on their diagnosis and projected length of treatment.

**Results:**

Twenty-eight patients (24 inpatient, 4 outpatient) were included in the study. The majority were uninsured (89%), homeless (64%), or had active intravenous drug use (IDU) (60%). Indications for use included SSTI (42.9%), bacteremia (64.3%), and OM (42.6%). Clinical failure was observed in 4 (14%), 1 (3.5%), and 2 (7.1%) patients at 30, 60, or 90 days (respectively). Nonadherence to medical recommendations, lack of source control, and ongoing IDU increased risk of returning to the hospital. Dalbavancin use saved a total of 381 days of inpatient/rehab/facility stay.

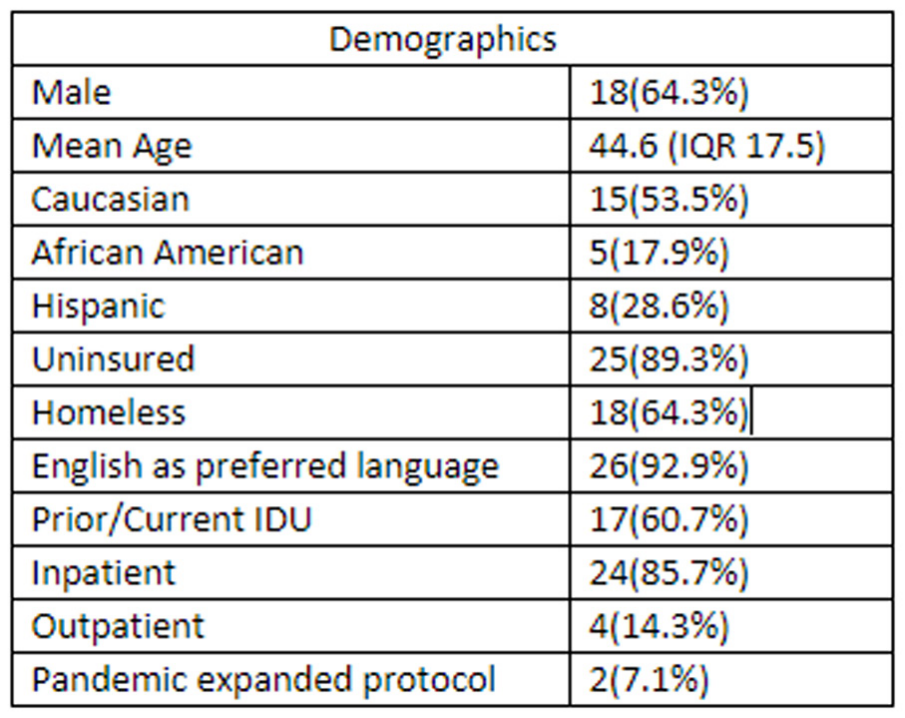

Baseline Characteristics of Patients

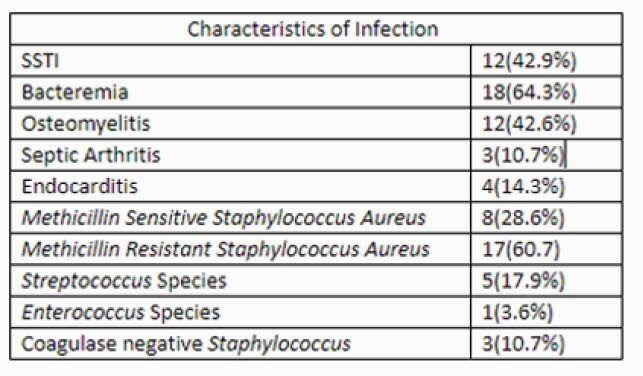

Types of Infections and Microbiology

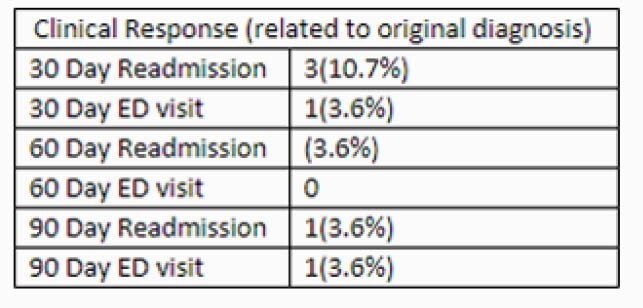

ED Visit or Readmissions at 30, 60, or 90 Days

**Conclusion:**

Dalbavancin showed similar rates of success with improved length of stay and cost savings. The use of long acting lipoglycopeptides are desirable alternatives to traditional OPAT for patients that otherwise would not qualify for OPAT or desire less hospital contact.

**Disclosures:**

**All Authors**: No reported disclosures

